# 

**Published:** 1910-10-22

**Authors:** 


					Appointments and Resignations.
Mr. Percy Nichol Allmax, L.R.C.S. and P. (Dublin),
has been appointed house-surgeon at the Herefordshire
General Hospital.
Mr. Kenneth Eckenstein, M.B., B.S. Lond., M.R.C.S.,
L.R.C.P., has been appointed assistant pathologist to the
National Hospital for Diseases of the Heart, Soho
Square, W.
The following appointments have been made at the
Norfolk and Norwich Hospital : Dr. W. L. Holyoak to
be house-surgeon, in place of Mr. W. A. Watson, re-
signed ; Mr. W. A. Watson to be house-physician, in place
of Mr. S. Trevor Davies, resigned; Mr. H. E. K. Frety
to be assistant house-surgeon, in place of Mr. C. C.
Holman, resigned; Mr. G. P. C. Claridge to fill the
newly created post of hon. pathologist and bacteriologist.
Obituary.
Dr. John Hewetson, of Riverside, California, has
died at Victoria, British Columbia.
The death is reported of Deputy Surgeon-General John.
Martin Hyde, A.M.D., in his eighty-firet year. He took
his M.R.C.S. in 1854, and after seeing service in various
parts of the world was placed upon the Retired List of
Army Medical Officers.
Dr. David Halket Stirling has died at KinnoulL
Cottage, Perth, in his eighty-second year. He studied
at Edinburgh, and became a licentiate of the Royal Col-
lege of Surgeons there in 1853. It was also at Edinburgh.
University that he obtained the degree of M.D. at the
same date.
The death is reported at Cheam, Surrey, of Dr. Jameson.
John Macan, formerly of Rockhampton, Queensland, aged
sixty-five. He became M.R.C.S. in 1870 and L.M. ia
1878, studying at Dublin, which originally was his home-
Re then pr-ssed to Cambridge, where he graduated M.D.r
becoming also an M.A. of that University.
Mr. Alfred Earnshaw Hewer, of Blackall, Queens-
land, has died at Hampstead, aged forty-five. He;
took his diploma in 1888, and graduated M.B., B.S., at
Cambridge in the following year. He was also a brother of
the " Meteor " Masonic Lodge, Longreach. He had been,
surgeon at Blackall Hospital and Government medical
officer at Blackall.
The death is announced at his country house, Lasting-
ham, Yorkshire, of Dr. Sydney Ringer, M.D., F.R.S., aged
seventy-six. He took his diploma in "1859, and the L.S.A.
in the same year, becoming in 1860 M.B. of London.
University. His many appointments included those of
resident medical officer, professor of materia medica, Holme.
Professor of Clinical Medicine, and consulting physician,
at University College Hospital. He had also been an.
examiner in materia medica at the University of London:
and at the Royal College of Physicians, as well as ail
assistant physician and medical registrar at the Children's
Hospital, Great Ormond Street, and clinical assistant at
the Brompton Consumption Hospital. He was an autho-
rity on the use of drugs, and his " Handbook on Thera-
peutics," now in its thirteenth edition, is a standard
work on the subject. He was a voluminous writer in his-
younger days, and "when he was only twenty-five years,
of age contributed a paper on ' Sound' to the Royal
Society."
Professor Ftjlgence Raymond, member of the French.
Academy of Medicine and Commander of the Legion of
Honour, died on September 29 near Poitiers, of heart
disease. Born on the same day of September 1844, he at.
first studied veterinary surgery, and soon his talent sig-
nalled him out for appointment as chief of the depart-
ment of anatomy and physiology at the Veterinary SchooL
of Alfort. At the age of twenty-three, however, he
turned his attention to medicine, and after passing the-
examinations for the " baccalaureat," he entered the School
of Medicine at Paris. In 1872 he obtained the post of " in-
terne," and in 1875 received the gold medal for a thesis on
" Hemichorea Hemiansesthesia and the Symptomatic-
Tremors." Two years later he was appointed " Chef de
Clinique " to the faculty, and the following year saw his
appointment as physician to the hospitals. In 1880 he
became joint professor. He quickly specialised in neurology
and mental diseases, and published in late years numerous-
works on these subjects. In 1894 he was elected to succeed:
his old master Charcot as professor at the Salpetriere. It
was only last year that the University of Oxford conferred
upon him the degree of Doctor of Science Honoris Causa..
The new Leicester Amateur Operatic Society has arranged
to give the proceeds of two performances of " The Lord
Protector," a comic opera, the libretto and lyrics of which
have been written by a Leicester journalist, Mr. C. J.
Tousley, and the music composed by Mr. C. E. Cowlrick,
the Society's musical director, to the funds of the Leicester
Infirmary. The performance will be given at the Temper-
ance Hall, Leicester, on December 1 and 2.
November being the anniversary of the opening of the
Jubilee Extension buildings of the National Hospital for
the Paralysed and Epileptic, Sir Frederick MacMillan,
chairman of the board of management, is appealing for
funds. The need of the hospital is great owing to the
scarcity of legacies, particularly during the past twelve
months. "Unless," says Sir Frederick, "we can provide
?5,000 before the end of this year we shall be faced with the
painful necessity of closing some of our wards."
120 THE HOSPITAL October 22, 1910.
Gifts and Bequests.
Mr. Daniel Hack, of With dean, Sussex, has left ?50 to
the Brighton Homoeopathic Dispensary.
The Treasurer of St. Bartholomew's Hospital acknow-
ledges the following further contribution towards the
special fund : Mr. George Acton Davis, ?100.
Mr. William Howard Durrant, of Ellery Court,
Upper Norwood, Australian merchant, who left estate
valued at ?72,810, has bequeathed ?250 to the British
Home and Hospital for Incurables, Streatham.
Mr. Edward Matyear, of Crabtree Farm, Fulham, the
last of Fulham's market gardeners, has bequeathed ?500
to the West London Hospital, Hammersmith, and the
residue of his estate to King Edward's Hospital Fund for
London.
The Rev. Henry Thornhill Morgan, late Vicar of St.
Peter in Eastgate with St. Margaret, Lincoln, who left
estate valued at ?38,391 gross, has bequeathed ?500 to the
East Derbyshire Hospital, Chesterfield, and ?100 to the
Royal Berkshire County Hospital, Reading.
The Hon. Robert Torrens O'Neill, of Tullymore
Lodge, Broughshane, county Antrim, Conservative M.P.
for Mid-Antrim from 1885 until the last General Election,
aged sixty-five, third eon of the first Baron O'Neill, who
left personal estate in the United Kingdom valued at
?39,848, has bequeathed ?500 to the Belfast Hospital
for Sick Children.
Mrs. Isabel Mary Lowe, of Palace Gate, Kensington,
has bequeathed ?1,000 each to the Nursing Branch of the
London Bible Women's and Nurses' Mission, the British
Medical Benevolent Fund (to be invested in an annuity
to be called the "Dr. John Lowe Annuity"), and the
Medical Mission Branch of the London Missionary Society
(in memory of her sister, Sarah Clayton Rawson), and
?500 to the Medical Mission Branch of the S.P.C.K. (in
memory of her husband).
The following contributions have been received recently
by the Treasurer of the Leicester Infirmary : Moira and
Donisthorpe Parade, ?35; Thorpe Satchville Church,
?26 16s. lOd.; Lutterworth Church, ?21 2s. 6d.; and
Broughton Astley Church, ?20 13s.; together with ?50
from the Ellistown Collieries, Ltd., while the following
sums over a hundred pounds have been sent by the work-
people of Messrs W. Tyler, Sons & Co., ?188 18s. 4d.;
Messrs. Walker, Hempson and Stevens, ?132 0s. lOd.;
Messrs. Fielding and Johnson, ?120 13s. 3d.; and Messrs.
Leavesley and North, ?105.
Jottings.
The Mayor of St. Pancras (Councillor J. T. Bryan) is
appealing for funds in aid of the University College Hos-
pital, Gower Street, W.C. A dinner will take place in
this connection at the Hotel Great Central, Marylebone
Road, on Wednesday, October 26, at 7.30 p.m. Tickets
for the dinner may be obtained from Mr. C. H. F. Bar-
rett (Town Clerk), who is acting as lion, secretary.
At a recent meeting of the King Edward Memorial
Committee in Birmingham, Mr. J. R. H. Smyth reported
that of the total amount subscribed (?28,480) ?22,890
was assigned for a new children's hospital. With re-
ference to the memorial hospital, Mr. H. K. Beale stated
that ?50,000 would be required to carry out the ideas of
the committee, which was instructed to confer as to erect-
ing the new hospital on land adjoining the Children's
Convalescent Home at Moseley.
Since Hospital Saturday began in 1873 the fund has
distributed ?517,106 among about 200 institutions. One
of the first letters opened last Saturday contained a
cheque for ?384, subscribed by members of the Stock
Exchange. The ordinary weekly collections in the work-
shops, &c.?the main income?amounted on October 8 to
?17,832, as compared with ?15,625 at the corresponding
period of last year. The annual dinner has been fixed
provisionally for February 11.
TO HOSPITAL SECRETARIES AND OTHERS.
We invite contributions from any of our readers, and
shall be glad to pay, according to length, for items of news
for the institutional section (including appointments, resig-
nations, gifts, etc.) which we may publish. All payments
for manuscripts used are made as early as possible after the
beginning of each quarter.
LEAGUE OF MERCY MEETINGS FOR THE WEEK.
Monday, October 24.?Mid-Essex District : Hon. Mrs. A.
Greville, At Home, Danbury Park, Chelmsford, 3 p.m.
Tuesday, October 25.?South St. Pancras District: Mrs.
Sykes At Home, 40 Camden Square, N.W., 4 p.m.
Wednesday, October 26.?Maldon District : Mrs. Price
At Home, Mark's Hall, Coggeehall, 2.45 p.m. ; and
Mile End District: Concert at the People's Palace,
organised by Mrs. Sidney Straus, 8 p.m.
Friday, October 28.?Berkshire District: Grand Morning
Concert by the Berks Symphony Orchestra, Drill Hall,
Wokingham, 3 p.m.
BUSINESS NOTICES.
Annual Subscriptions.
The rate of annual subscriptions to The
Hospital, either direct from the Office or through
agents, is: ?
For the United Kingdom  15s. a year.
For the Colonies and Abroad ... 17s. ,,
For the United Kingdom (with
Insurance Policy)  ?1 ,,
inclusive of postage in each case.
Letters relating to the Publishing, Sale and
Advertisement Departments must be addressed to
the Manager (not to the Editor): ?
The Manager,
The Hospital Building,
28 and 29 Southampton Street,
Strand, London, W.C.
The Relaxations of Medical Men.
We shall also be glad to pay for accepted contri-
butions, from any member of the profession, on the
subject of the relaxations of practitioners. This
opens up a wide field, as it includes natural history,
photography, sport, indoor recreations, and motor-
ing. Whenever possible, original illustrations and
photographs should be sent with the MS.
Suggestions Invited.
The Editor will welcome suggestions for the estab-
lishment of any new section in The Hospital, and
will be glad to supply information on any subject of
interest or importance to members of the profession
in any part of the world.
THE SCIENTIFIC PRESS PUBLICATIONS
MEDICAL HISTORY FROM THE I THE CONSUMPTIVE WORKING MAN
EARLIEST TIMES.
By E. T. Withington, M.A., M.B. Oxon. Demy 8vo.
over 400 pages, with 2 Plates, cloth gilt, 12s. 6d. net;
12b. lOd. post free.
PHOTOMICROGRAPHY.
By Edmund J. Spitta, L.R.C.P. Lond.; M.R.C.S. Eng.,
F.R.A.S. Demy 4to. with 41 Half-tone Reproductions
from Original Negatives and 63 Illustrations in the Text,
cloth gilt, 12s.
THE SCHOTT TREATMENT FOR
CHRONIC HEART DISEASES.
By Richard Greene, F.R.C.P. Ed. Second Edition,
Revised, demy 8vo. paper cover, 6d.
THE MENOPAUSE AND ITS DISORDERS.
With Chapters on Menstruation. By A. D. Leith
Napier, M.D., M.R.C.P. Royal 8vo. cloth gilt, illustrated
by a series of Original Photo-micrographs and many
Illustrations in the Text, 7s. 6d. net; 7s. lid. post free.
What can Sanatoria do for him ?
By Noel Dean Bardswell, M.D., Medical Superintendent,
King Edward VII. Sanatorium, Midhurst. Demy 8vo.
illustrated, cloth, 10s. 6d. net; 10s. lOd. post free.
SANATORIUM TREATMENT OF
CONSUMPTION.
By T. N. Kelynack, M.D., M.R.C.P. Crown 8vo. limp
cloth cover, 6d. net; 7d. post free.
THE SELECTION OF CONSUMPTIVE
CASES for SANATORIUM TREATMENT.
By T. N. Kelynack, M.D., M.R.C.P. Crown 8vo. limp
cloth cover, 6d. net; 7d. post free.
CLINICAL DIAGNOSIS:
A Practical Handbook of Chemical and Microscopical
Methods. By W. G-. Aitchison Robertson, M.D.,
F.R.C.P. Edin. Fcap. 8vo. profusely Illustrated, cloth
gilt, 3s. 6d. net; 3s. 9d. post free.
OBTAINABLE OF ALL BOOKSELLERS, OR OF
THE SCIENTIFIC PRESS, LTD., Medical and Scientific Publishers,

				

